# Automated medical chart review for breast cancer outcomes research: a novel natural language processing extraction system

**DOI:** 10.1186/s12874-022-01583-z

**Published:** 2022-05-12

**Authors:** Yifu Chen, Lucy Hao, Vito Z. Zou, Zsuzsanna Hollander, Raymond T. Ng, Kathryn V. Isaac

**Affiliations:** 1 Department of Computer Science, University of British Columbia, Faculty of Science, 201-2366 Main Mall, Vancouver, BC V6T 1Z4 Canada; 2 Prevention of Organ Failure (PROOF) Centre of Excellence, 1190 Hornby Street, Vancouver, BC V6Z 2K5 Canada; 3 Department of Surgery, University of British Columbia, Faculty of Medicine, 2221 Wesbrook Mall, Vancouver, BC V5Z 1M9 Canada

**Keywords:** Natural language processing, Breast cancer, Health data

## Abstract

**Background:**

Manually extracted data points from health records are collated on an institutional, provincial, and national level to facilitate clinical research. However, the labour-intensive clinical chart review process puts an increasing burden on healthcare system budgets. Therefore, an automated information extraction system is needed to ensure the timeliness and scalability of research data.

**Methods:**

We used a dataset of 100 synoptic operative and 100 pathology reports, evenly split into 50 reports in training and test sets for each report type. The training set guided our development of a Natural Language Processing (NLP) extraction pipeline system, which accepts scanned images of operative and pathology reports. The system uses a combination of rule-based and transfer learning methods to extract numeric encodings from text. We also developed visualization tools to compare the manual and automated extractions. The code for this paper was made available on GitHub.

**Results:**

A test set of 50 operative and 50 pathology reports were used to evaluate the extraction accuracies of the NLP pipeline. Gold standard, defined as manual extraction by expert reviewers, yielded accuracies of 90.5% for operative reports and 96.0% for pathology reports, while the NLP system achieved overall 91.9% (operative) and 95.4% (pathology) accuracy. The pipeline successfully extracted outcomes data pertinent to breast cancer tumor characteristics (e.g. presence of invasive carcinoma, size, histologic type), prognostic factors (e.g. number of lymph nodes with micro-metastases and macro-metastases, pathologic stage), and treatment-related variables (e.g. margins, neo-adjuvant treatment, surgical indication) with high accuracy. Out of the 48 variables across operative and pathology codebooks, NLP yielded 43 variables with F-scores of at least 0.90; in comparison, a trained human annotator yielded 44 variables with F-scores of at least 0.90.

**Conclusions:**

The NLP system achieves near-human-level accuracy in both operative and pathology reports using a minimal curated dataset. This system uniquely provides a robust solution for transparent, adaptable, and scalable automation of data extraction from patient health records. It may serve to advance breast cancer clinical research by facilitating collection of vast amounts of valuable health data at a population level.

**Supplementary Information:**

The online version contains supplementary material available at 10.1186/s12874-022-01583-z.

## Background

Cancer burden is an important challenge in healthcare due to the growing incidence, morbidity, and cost [1–4]. Breast cancer is the most common cancer in women, with over 1.6 million women diagnosed per year globally [5]. Over the last 2 decades, translation of clinical research into practice has led to significant improvement in breast cancer survival and quality of life. Advances in clinical research have relied on population-based data, including cancer staging and clinical outcomes data collected in regional databases and collated in national databases. As the number of patients with cancer increases, a parallel growth has occurred in recorded health data. Electronic formats of medical records are becoming ubiquitous across health care systems [6]. Electronic health records (EHRs) are a fruitful source of information that can be utilized to garner novel understanding of a disease’s natural history [7], treatment responses, and prognosis [8–10] to guide and advance clinical breast cancer research [11–15]. National research and quality improvement registries aim to efficiently collate population-based health data [16, 17]. Across North America, with a few potential exceptions [12, 13], the bulk of this valuable EHR information is currently being extracted by manual review, which significantly limits timeliness, scalability, and research due to high costs.

With exponential growth of health data and constrained healthcare budgets, the volume of work for data extraction can rapidly exceed capacity, leading to time delays, and restrictions on the scope of variables extracted. This challenge is amplified by the increased volume and complexity of data rising with the synchronous expansion of cancer cases and clinical knowledge pertinent for diagnosis, management, and prognosis [18]. Regional cancer registries, including some institutions, are mandated to report on cancer outcomes and support oncologic population health research. With finite resources in the health care system for cancer surveillance and monitoring, regional cancer databases must limit and prioritize the extraction of specific variables of known relevance to treatment planning and prognosis. In response to strained resources, the scope of information and timeliness of data input into cancer databases have suffered and are limited as opposed to expanding. This inefficiency hinders innovative exploratory clinical research using big data [19].

Computational methods are rapidly being developed and translated into healthcare to automate and expedite data extraction from EHRs. Natural Language Processing (NLP) utilizes computational methods to analyze language, where text and speech data inputs are used for processing and capturing meaning from words. NLP algorithms are most commonly tasked to extract text and recognize specific entities. Current methods for text processing in the cancer domain include three relevant categories of NLP strategies: named entity recognition (NER), information extraction (IE), and text classification (TC). NER identifies terms and classifies these according to predefined categories, relying on dictionaries of biomedical terms, for e.g. Unified Medical Language System (UMLS) metathesaurus used via MetaMap to obtain terms annotated to entities [13]. An important challenge with reliance on standardized dictionaries is the common existence of term variability. Terms may often not be found in the source dictionaries because of synonyms, acronyms, abbreviations, or idiosyncrasies (like grammatical errors) which requires additional strategies [20]. IE methods identify predefined facts and relationships of interest, often using NER and additional modelling using regular expression pattern-matching rules and negation rules. This fine-tuning approach is highly reliable for IE of valuable clinical information specific to a cancer type (i.e. Nottingham score for breast cancer), especially when records have structural conformity [21]. TC extends the benefits of IE to infer information that is not explicitly stated, but derived by predefined rules, whereby a cancer can be classified into a predefined category according to an expert-derived program of rules for inductive reasoning [22]. With the hierarchical building of IE and TC strategies on foundational methods of NER, it is critical to have high accuracy in the lexicon. Any baseline errors or compromise in the entities upon which the final algorithm is built will limit the scalability, accuracy, and generalizability of the algorithm.

Recent advances in deep learning (DL) methods have yielded new breakthroughs as well as challenges. While traditional algorithms rely on explicit rules engineered by humans, DL methods autonomously formulate inference parameters by learning from large datasets. The robustness of a DL model relies on the size and quality of its training datasets. For example, state-of-the-art biomedical language models [23–25] were trained on billions of words from Wikipedia and PubMed. Although DL models can be fine-tuned to perform a wide range of downstream tasks, their inherent dependence on massive volumes of data poses new challenges to healthcare adopters who lack access to large-scale EHR datasets [26]. Even with sufficient training data, the “black box” unexplainable nature of DL models often concern healthcare stakeholders [27].

To address the challenges, this study aimed to develop a fully customized NLP extraction system to automate extraction of clinically relevant diagnostic, treatment, and prognostic outcomes data into a population-based regional cancer database. Given the real-world limitations of massive annotated EHRs, we leveraged an optimal use of NLP computational strategies and word embeddings trained on open access biomedical datasets. While the system does not use DL methods directly, we do use pre-trained embedding models to develop the algorithm using a small subset of annotated clinical cases. This de novo rule-based, transparent, and explainable algorithm is user-friendly, adaptable, and scalable to expand volumes and variety of information, providing oncology clinical researchers with a tool to expedite data collection, a laborious task inherent in most research endeavors.

## Methods

### Overview

**Fig. 1:**
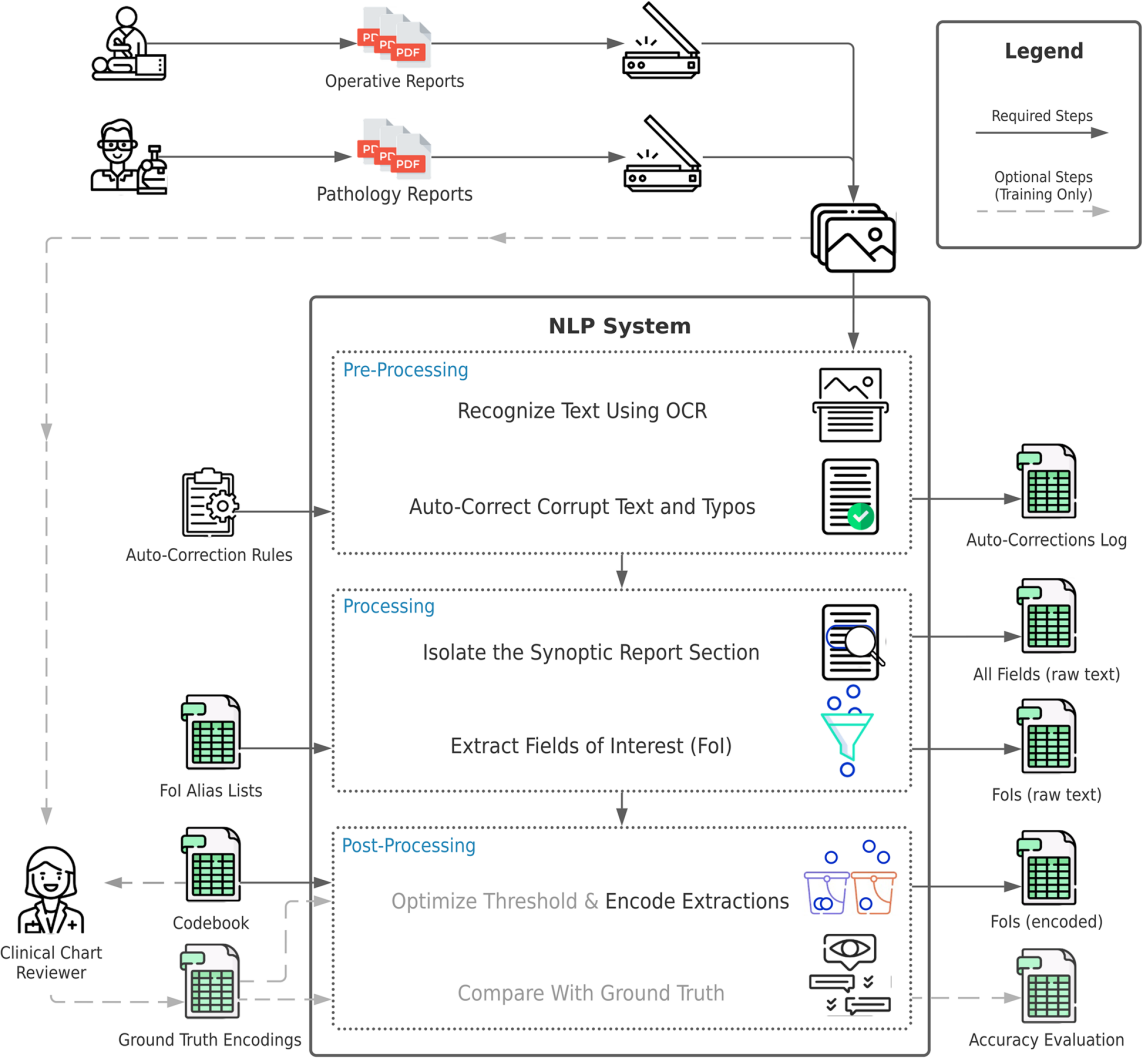
Overview of the NLP system design, input, and outputs

The NLP pipeline was developed to automate the extraction of salient breast cancer outcomes designated for automated extraction from breast cancer patient EHRs, specifically from structured operative and pathology reports. Fig. 1 displays a high-level summary of the study design, datasets, and the NLP system.

The operative and pathology EHRs contain a standardized synoptic section, and we leveraged its structural conformity to develop the customized NLP algorithm. Using optimized EHR formats, the NLP algorithm first runs a uniquely generated “pattern matcher” of custom rules to extract text phrases corresponding to each variable. We then encode the extractions with a biomedical word embedding model pre-trained on large-scale biomedical datasets [28]. The algorithm development was based on a curated sample of 100 records, with 50 pathology and 50 operative reports. The testing was performed on an additional 50 pathology and 50 operative records. Institutional study ethics approval was obtained with waiver of consent.

### Data sources

The training and test datasets were derived from a cohort of consecutive patients treated at the University of British Columbia between January 1st 2015 to 2021 for breast cancer resection and reconstruction. Patients received their operative intervention at one institution by a group of 7 clinicians. Operative and pathology report text and speech inputs were generated by a heterogenous group of over 20 medical experts in breast cancer care, including surgical oncologists and pathologists. All reports contain a structured, Synoptic Report section upon which we applied NLP. Patients were excluded if the reports were inaccessible, were archived in error, or contained addendums to prior reports. All operative and pathology reports were annotated to capture salient outcomes determined a priori, defined and outlined in a codebook. This codebook was used by both the human reviewers and the NLP system. Each report was independently reviewed by human data extractors with expert medical knowledge and experience in medical record review. Salient outcomes were manually extracted and encoded into curated database. To enable comparison of data quality between human and algorithm accuracies, human reviewers were selected with differing baseline experiences, and a third expert reviewer assessed and confirmed the data quality.

The NLP system was developed using a training dataset of 50 operative and 50 pathology reports derived from the study cohort. The NLP system was evaluated using a separate unseen test dataset of 50 operative and 50 pathology reports derived from the same study cohort. The training and test datasets had the same inclusion and exclusion criteria. There was no overlap between patient sources of the training and test sets.

### Variables

Operative outcomes included data on indication, diagnosis, laterality, procedure type, lymph nodes removal, incision type, wire localization, and immediate reconstruction methods. Pathology outcomes included details of tumor diagnosis, size, margins of resection, Nottingham score, focality, number of positive lymph nodes, and pathologic stage. Variable types are defined and reported in the codebook (Supplemental Table 1).

### Pipeline development

The extraction pipeline combines novel custom algorithms with peer-reviewed biomedical text processing [28, 29], including NER and TC strategies. The pipeline algorithm may be viewed as having three-steps: pre-processing, processing, and post-processing (Fig. 1). Transparency, adaptability, and data quality assurance mechanisms are embedded throughout NLP system to optimize integration of human and artificial intelligence.

Pre-processing modules were used to convert the input documents into correct formats. Two methods were employed to pre-process PDF formats depending on their content. For reports that mainly contain alphabetical values (operative reports), an open-source Optical Character Recognition (OCR) tool, pytesseract [29], was used to transform scanned PDF images to text. Pytesseract offers significant accuracy gain compared to Adobe Acrobat on reports with mainly alphabetical values. However, pytesseract often misinterprets numerical values, thus Adobe Acrobat’s OCR tool was applied to pathology reports to improve the recognition of numerical values (e.g., tumour size, distance from margins, etc.). To increase the accuracy of these OCR tools, we developed auto-correction algorithms that address common OCR errors (e.g., word fragmentation). A Graphical User Interface displays the auto-corrected text for NLP system transparency and quality assurance, allowing human users to easily review and rectify any remaining OCR errors and re-run the system. These manual corrections were permanently integrated into the NLP system via persistent disk storage. The system is able to work fully autonomously on unseen EHRs because the most common OCR issues are repetitive (e.g., “DCIS” vs “DC1S”) and have already been addressed.

After pre-processing, the processing module uses a template-based pattern-matching algorithm to extract the targeted outcomes defined in the codebook, with each variable defined as a Field of Interest (FoI). We developed a regular expression pattern generator to search for the FoIs based on signpost phrases and position relative to the EHR template. Note, in contrast with hardcoded rule-based regular expressions, the generator is generic and can be expanded to extract additional new FoIs. When the system cannot find a FoI, a custom search algorithm uses Levenshtein edit-distance methods [30] to find the most similar spelling candidate. The Levenshtein distance between two strings is the number of single-character edits required to turn one string into another. For example, the edit distance between “biopsy” and “biopsies” is 3. The extracted FoIs progress to the post-processing step in original raw text format to maintain data granularity of information and facilitate review.

The post-processing module encodes the extracted text into numeric labels. To ensure robustness in processing rare biomedical terms, we used a biomedical word embedding model, scispaCy, which was pre-trained on biomedical text [28]. Embeddings of words are numeric vector representations in high-dimensional space, such that semantically similar words are clustered together (Additional file 3). To encode the original text, we compare the cosine similarity between embeddings of codebook candidates and extracted text with customized thresholds (Additional file 4). We choose the encoding with the highest similarity to the EHR text. If none of the encoding candidates scored above the threshold, the actual text value was extracted.

Following NLP pipeline development, errors in the NLP pipeline extraction dataset outcomes were examined in preliminary analysis. The FoIs with the accuracies below 80% were analyzed and strategies employed to correct these extraction failures (Supplemental Table 2).

For user-friendly adaptation and evolution of the NLP pipeline, new reports and FoIs added are rapidly evaluated using a visualization accuracy tool which displays the comparison of results between the NLP pipeline and human derived data.

We have released the codebase for the NLP system along with a usage guide at: https://github.com/chen-yifu/EMR_pipeline .

### Statistical analysis

Clinical characteristics of training and test sets were summarized with descriptive statistics in Table 1.

**Table 1:** Diagnostic and treatment characteristics in operative and pathology report cohorts. SD; standard deviation, LN; lymph node

**Pathology Reports**	Training Cohort (*n* = 50)	Validation Cohort (*n* = 50)
Laterality		
Unilateral	45 (90%)	49 (98%)
Bilateral	5 (10%)	1 (2%)
Cancer Type		
Invasive	42 (76.4%)	41 (80.4%)
Non-Invasive	13 (23.6%)	10 (19.6%)
Margins		
Positive	10 (18.2%)	9 (17.6%)
Negative	45 (81.8%)	42 (82.4%)
Lymph Nodes		
Avg. LNs Examined (SD)	4.6 (4.3)	4.3 (3.4)
Micro/Macro Metastasis	16 (29.1%)	13 (25.5%)
Extranodal Extension	8 (14.5%)	3 (5.9%)
Pathologic Diagnosis		
Avg. Number of Foci (SD)	1.9 (2.0)	2.0 (2.2)
Avg. Nottingham Score (SD)	6.3 (1.6)	6.7 (1.6)
Avg. Tumour Size in mm. (SD)	28.2 (28.8)	27.9 (20.0)
Lymphovascular Invasion	11 (20%)	10 (19.6%)
**Operative Reports**	Training Cohort (*n* = 50)	Validation Cohort (*n* = 50)
Laterality		
Unilateral	48 (96%)	49 (98%)
Bilateral	2 (4%)	1 (2%)
Procedure Type		
Lumpectomy	19 (36.5%)	16 (31.4%)
Nipple-Sparing Mastectomy	15 (28.9%)	21 (41.2%)
Skin-Sparing Mastectomy	16 (30.7%)	14 (27.5%)
Total Mastectomy	2 (3.8%)	0
Neoadjuvant Treatment		
Chemotherapy	4 (7.7%)	14 (27.5%)
None	48 (92.3%)	37 (72.5%)
Immediate Reconstruction		
Mentioned	50 (96.2%)	45 (88.2%)
Not Mentioned	2 (3.8%)	6 (11.8%)
Axillary Surgery		
Sentinel LN Biopsy	40 (76.9%)	38 (74.5%)
Axillary LN Dissection	4 (7.7%)	5 (9.8%)
None	8 (15.4%)	8 (15.6%)

To evaluate the performance of the NLP pipeline against human manual extraction, a ground truth (GT) or “gold standard” of the manual extraction was required. Human data extractors consisted of two senior medical students and third independent review was conducted by academic breast surgeon. GT was selected by identifying the human extracted dataset with the highest accuracy established by a third independent human reviewer who compared the two human-derived datasets and original reports. GT represents the human-derived dataset with the highest accuracy, defined as the number of extracted variables matching the original reports. The inter-rater agreement between two human-extracted datasets was measured.

Detailed performance metrics for each Field of Interest (FoI) were calculated by the following formulae:

$${\displaystyle \begin{array}{c}\mathrm{Accuracy}=\mathrm{Correct}\ \mathrm{predictions}\div \mathrm{Total}\ \mathrm{predictions}=\left(\mathrm{TP}+\mathrm{TN}\right)\div \left(\mathrm{TP}+\mathrm{TN}+\mathrm{FP}+\mathrm{FN}\right)\\ {}\mathrm{Precision}=\mathrm{True}\ \mathrm{positive}\div \mathrm{Predicted}\ \mathrm{positive}=\mathrm{TP}\div \left(\mathrm{TP}+\mathrm{FP}\right)\\ {}\begin{array}{c}\mathrm{Recall}=\mathrm{True}\ \mathrm{positive}\div \mathrm{Actual}\ \mathrm{positive}=\mathrm{TP}\div \left(\mathrm{TP}+\mathrm{FN}\right)\\ {}\mathrm{F}-\mathrm{Score}=2\times \left(\left(\mathrm{Precision}+\mathrm{Recall}\right)\div \left(\mathrm{Precision}+\mathrm{Recall}\right)\right)\\ {}\mathrm{Cohen}'\mathrm{s}\ \mathrm{Kappa}=\left({\mathrm{p}}_{\mathrm{o}}-{\mathrm{p}}_{\mathrm{e}}\right)\div \left(1-{\mathrm{p}}_{\mathrm{e}}\right)\end{array}\end{array}}$$

Overall NLP pipeline performance was compared to the GT, defined as the most accurate human extracted dataset. Study size was defined by the size of data set required to develop an algorithm with over 90% accuracy in the training set. To minimize bias of datasets, consecutive cases were considered for inclusion.

## Results

The NLP system was developed to automate the collection of 48 salient outcomes based on 2607 training data points from 100 EHRs and was evaluated using the test dataset of another 100 EHRs. Clinical characteristics of the study cohort in each training and test set is displayed in Table 1. Majority of the targeted outcomes (FoI) were derived from pathology reports (*n* = 37, 76%), as compared to operative reports (*n* = 11, 24%).

### NLP system development with training cohort

The highest accuracy human-derived dataset, defined as GT, was used for development of NLP pipeline and performance assessments in the training cohort. The NLP pipeline achieved 93.3 and 96.1% overall accuracy as compared to the GT. In the training cohort, the NLP pipeline outperformed the second, less accurate, human-derived dataset on both the operative and pathology training cohorts.

### NLP system performance evaluation with test cohort

To evaluate the NLP pipeline’s performance, it was deployed on a test cohort which is previously completely unseen by the NLP pipeline and its programmers. Detailed performance metrics for each FoI are shown in Tables 2 and 3. The NLP pipeline achieved an overall 91.9% accuracy for the operative reports and 95.4% accuracy for the pathology reports as compared to GT. A precision score of 0.95 was achieved for operative FoIs and precision score of 0.97 for pathology FoIs. Recall of 0.97 was achieved on both operative and pathology FoIs. F-scores of 0.96 and 0.97 was achieved on operative and pathology FoIs, respectively. Of the 11 FoIs derived from the operative reports, all achieved F-scores and recall of at least 0.90 and nearly all (10 of 11) have precision of 0.90. From the pathology reports, most of the 37 FoIs have precisions (34 of 37), recall (32 of 37), and F-score (32 of 37) of at least 0.90. In summary, out of the 48 operative and pathology FoIs, 44 (92% of 48 FoIs), 43 (90%), and 43 (90%) FoIs have at least 0.90 precision, recall, and F-scores, respectively. At higher standards, 35 (73%), 41 (85%), and 38 (79%) FoIs have at least *0.95* precision, recall, or F-scores, respectively.

**Table 2:** Detailed accuracy metrics for the NLP system with respect to the ground truth (GT) in operative reports

Outcome variable	Accuracy	Precision	Recall	F-Score
Laterality	0.90	0.94	0.96	0.95
Surgical Indication	0.96	0.98	0.98	0.98
Pre-Operative Biopsy	0.96	1.00	0.96	0.98
Pre-Operative Diagnosis	0.96	0.98	0.98	0.98
Neoadjuvant Treatment	0.98	1.00	0.98	0.99
Breast Procedure	0.92	0.94	0.98	0.96
Immediate Reconstruction	0.92	0.94	0.98	0.96
Immediate Reconstruction Type	0.86	0.90	0.96	0.92
Wire Localization	0.88	0.92	0.96	0.94
Breast Incision Type	0.87	0.89	0.96	0.93
Axillary Surgery	0.90	0.96	0.94	0.95
**Overall**	**0.92**	**0.95**	**0.97**	**0.96**

**Table 3:** Detailed accuracy metrics for the NLP system with respect to the ground truth (GT) in pathology reports. DCIS; ductal carcinoma in situ, LN; lymph nodes

Outcome Variable	Accuracy	Precision	Recall	F-Score
Invasive Carcinoma	1.00	1.00	1.00	1.00
Invasive Histologic Type	0.94	0.94	1.00	0.97
Nottingham Score	0.98	1.00	0.97	0.99
Glandular Differentiation	0.96	0.95	1.00	0.98
Nuclear Pleomorphism	0.98	0.98	1.00	0.99
Mitotic Rate	0.98	0.98	1.00	0.99
Histologic Grade	0.96	0.97	0.97	0.97
Tumour Size (mm)	0.92	0.95	0.95	0.95
Tumour Focality	1.00	1.00	1.00	1.00
# of Foci	0.98	1.00	0.97	0.99
Tumour Site	1.00	1.00	1.00	1.00
Lymphovascular Invasion	0.96	0.95	1.00	0.98
In situ Component	0.98	0.98	1.00	0.99
In situ Type	0.98	1.00	0.98	0.99
In situ Nuclear Grade	1.00	1.00	1.00	1.00
Necrosis	0.98	0.97	1.00	0.99
DCIS Extent	0.82	0.86	0.79	0.83
Architectural Patterns	1.00	1.00	1.00	1.00
Invasive Carcinoma Margins	0.94	0.93	1.00	0.97
Distance from Closest Margin	0.84	0.97	0.81	0.88
Closest Margin	0.90	0.97	0.89	0.93
DCIS Margins	0.78	0.78	1.00	0.88
Distance of DCIS from Closest Margin (mm)	0.86	0.92	0.81	0.86
Closest Margin DCIS	0.83	0.90	0.76	0.83
Total LN Examined	0.98	1.00	0.98	0.99
# Sentinel LN Examined	1.00	1.00	1.00	1.00
Micro/macro metastasis	0.88	0.87	1.00	0.93
# LN with Micro-metastasis	1.00	1.00	1.00	1.00
# LN with Macro-metastasis	1.00	1.00	1.00	1.00
Size of largest Macro-metastasis Deposit	0.98	1.00	0.91	0.95
Extranodal Extension	1.00	1.00	1.00	1.00
Extent (mm)	1.00	1.00	1.00	1.00
Invasive Tumour Size (mm)	0.94	0.95	0.97	0.96
# Sentinel Nodes Examined	0.96	0.95	1.00	0.97
# Micro-metastatic Nodes	1.00	1.00	1.00	1.00
# Macro-metastatic Nodes	1.00	1.00	1.00	1.00
Pathologic Stage	0.98	1.00	0.98	0.99
**Overall**	**0.95**	**0.97**	**0.97**	**0.97**

### Error analysis

For operative reports, NLP system incurred the greatest difficulty extracting the variables “incision type” (precision 0.89, recall 0.96, F-score 0.93), “immediate reconstruction type” (precision 0.89, recall 0.96, F-score 0.93), and wire localization (precision 0.92, recall 0.96, F-score 0.94) (Table 2). In the pathology reports, variables with the greatest inaccuracies were related to details of Ductal Carcinoma In Situ disease (DCIS) and surgical margins. Specifically, the NLP pipeline failed to reach 90% threshold of accuracy for DCIS margins positivity (precision 0.79, recall 1.00, F-score 0.88), DCIS extent (precision 0.86, recall 0.79, F-score 0.83), closest DCIS margin location (precision 0.90, recall 0.76, F-score 0.83), and closest invasive carcinoma margin distance (precision 0.91, recall 0.81, F-score 0.86) (Table 3). While the NLP pipeline was accurate in reporting the number of lymph nodes with metastases (precision 1.00, recall 1.00, F-score 1.00), the algorithm failed to correctly extract the presence or absence of micro or macro-metastases as a binary encoding (precision 0.87, recall 1.00, F-score 0.93).

Through an error analysis of the extractions, we found three main causes affecting performance. First, false negatives occurred when information was absent from the structured section of the report and was instead located in unstructured text, which is ignored by the NLP system and reviewed by the human annotator. Second, there exists differences in terminology used across cohorts. For example, most training pathology reports used “DCIS Extent” to indicate the “DCIS Extent” FoI, while “DCIS Estimated Size” was used in other test reports – resulting in false negatives. Third, the NLP system and human rarely disagreed on how to encode the same text. For example, an EHR reported the “Distance from Closest Margin” as “cannot be determined - greater than 10 mm.” The NLP and GT extracted “N/A” and “> 10”, respectively.

### Inter-annotator agreement of human-extracted datasets

Across operative reports, an inter-annotator agreement Cohen’s Kappa score of 0.50 was found (moderate agreement). Across pathology reports, a score of 0.90 was found (almost perfect agreement). As compared to GT, the second human dataset achieved 90.5 and 96.0% accuracy on the operative and pathology cohorts respectively. Greatest discrepancies were identified for operative report variables, with accuracy differences up to 61% for immediate reconstruction type (Table 2). Accuracy, precision, recall, and F-scores with respect to each FoI are shown in Tables 4 and 5. Across the 48 FoIs extracted by human, 43 (90% of FoIs), 48 (100%), and 44 (92%) FoIs have higher than 0.90 precision, recall, and F-scores respectively; 40 (86%), 38 (79%), 41 (85%) FoIs have higher than *0.95* precision, recall, and F-scores respectively.

**Table 4:** Detailed accuracy metrics for the human annotator with respect to the ground truth (GT) in operative reports. All scores were computed by averaging the metrics across training and test cohorts

Outcome variable	Accuracy	Precision	Recall	F-Score
Laterality	0.99	0.99	1.00	1.00
Surgical Indication	0.97	0.97	1.00	0.99
youPre-Operative Biopsy	0.98	0.98	1.00	0.99
Pre-Operative Diagnosis	0.93	0.93	1.00	0.97
Neoadjuvant Treatment	0.98	0.98	1.00	0.99
Breast Procedure	0.97	0.97	1.00	0.99
Immediate Reconstruction	0.89	0.70	1.00	0.80
Immediate Reconstruction Type	0.60	0.66	0.99	0.78
Wire Localization	0.90	0.76	0.99	0.84
Breast Incision Type	0.81	0.89	0.84	0.85
Axillary Surgery	0.96	0.96	1.00	0.98
**Overall**	**0.91**	**0.89**	**0.98**	**0.92**

**Table 5:** Detailed accuracy metrics for the human annotator with respect to the ground truth (GT) in pathology reports. DCIS; ductal carcinoma in situ, LN; lymph nodes. All scores were computed by averaging the metrics across training and test cohorts

Outcome Variable	Accuracy	Precision	Recall	F-Score
Invasive Carcinoma	0.98	1.00	0.98	0.99
Invasive Histologic Type	0.95	0.97	0.98	0.97
Nottingham Score	0.62	1.00	1.00	1.00
Glandular Differentiation	0.97	0.99	0.98	0.98
Nuclear Pleomorphism	0.97	0.98	0.98	0.98
Mitotic Rate	0.96	0.95	0.98	0.97
Histologic Grade	0.96	0.99	0.95	0.97
Tumour Size (mm)	0.98	0.96	0.98	0.97
Tumour Focality	0.96	0.97	0.98	0.97
# of Foci	0.96	0.98	0.97	0.97
Tumour Site	0.95	0.74	0.94	0.81
Lymphovascular Invasion	0.97	0.98	0.98	0.98
In situ Component	0.95	0.99	0.94	0.97
In situ Type	0.97	0.99	0.97	0.98
In situ Nuclear Grade	0.96	0.98	0.96	0.97
Necrosis	0.96	0.96	0.96	0.96
DCIS Extent	0.98	0.97	0.95	0.96
Architectural Patterns	0.96	0.93	0.95	0.94
Invasive Carcinoma Margins	0.96	0.97	0.98	0.97
Distance from Closest Margin	0.97	0.99	0.96	0.97
Closest Margin	0.97	1.00	0.96	0.98
DCIS Margins	0.94	0.94	0.97	0.95
Distance of DCIS from Closest Margin (mm)	0.95	0.99	0.94	0.96
Closest Margin DCIS	0.97	1.00	0.94	0.97
Total LN Examined	0.98	1.00	0.98	0.99
# Sentinel LN Examined	0.98	1.00	0.98	0.99
Micro/macro metastasis	0.98	1.00	0.98	0.99
# LN with Micro-metastasis	0.98	1.00	0.96	0.98
# LN with Macro-metastasis	0.98	1.00	0.96	0.98
Size of largest Macro-metastasis Deposit	0.98	1.00	0.95	0.98
Extranodal Extension	0.98	1.00	0.94	0.97
Extent (mm)	0.98	1.00	0.90	0.95
Invasive Tumour Size (mm)	0.97	1.00	0.97	0.98
# Sentinel Nodes Examined	0.96	0.96	0.96	0.96
# Micro-metastatic Nodes	0.98	1.00	0.95	0.98
# Macro-metastatic Nodes	0.97	1.00	0.93	0.96
Pathologic Stage	0.98	1.00	0.98	0.99
**Overall**	**0.96**	**0.98**	**0.96**	**0.97**

## Discussion

**Fig. 2:**
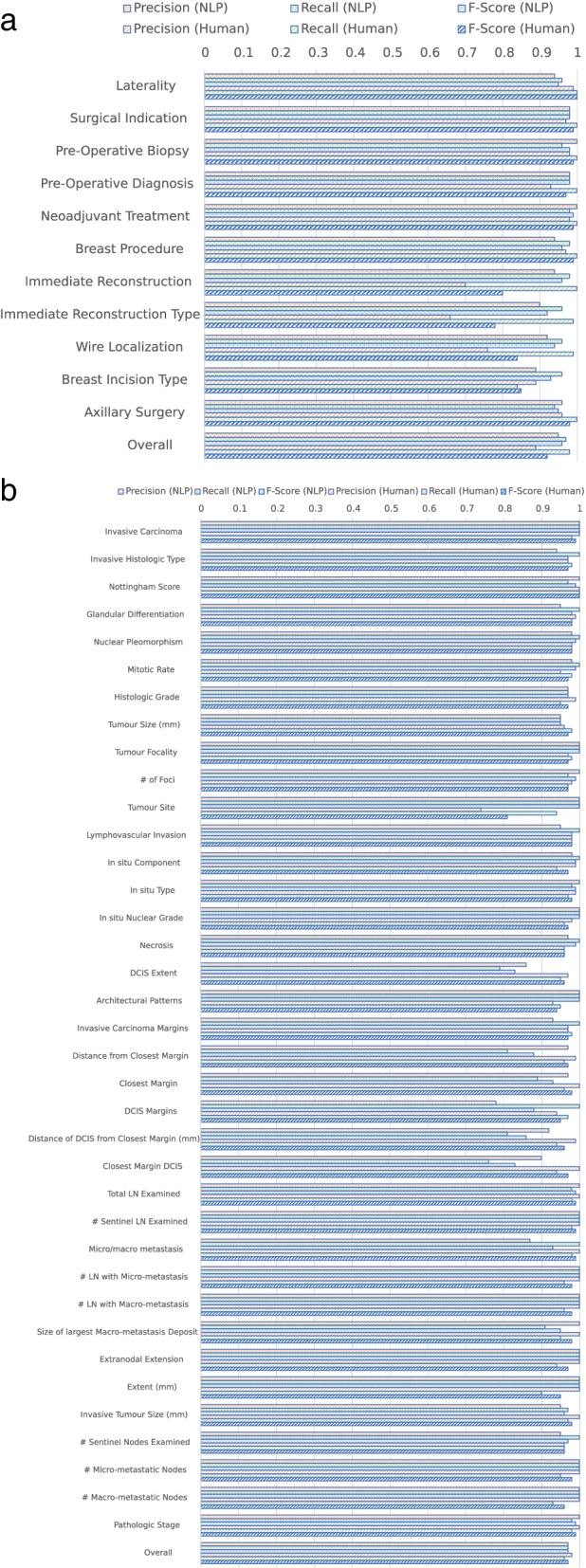
**a** NLP versus the second human reviewer as compared to the GT in operative reports. **b** NLP versus the second human reviewer as compared to the GT in pathology reports

This study describes the development and evaluation of a customizable automated data extraction NLP pipeline for breast cancer outcomes data using a minimal size cohort of patients. Using a combination of rule-based (pattern-matching, auto-correction) and statistics-based (pre-trained biomedical word embeddings) methods, the developed NLP algorithm is robust, transparent, and adaptable. In the test cohort, the NLP pipeline did not outperform the second, less accurate, human-derived dataset for either the pathology or operative reports (Fig. 2a, b). Notably, the human reviewer outperformed the NLP pipeline in pathology reports containing both invasive and in situ disease, with interpretation required for DCIS margins (NLP 78% accuracy) and extent of disease (NLP 82% accuracy). Nevertheless, the NLP pipeline achieved near-human-level accuracy across most of the 48 targeted outcomes relevant to clinical outcomes research. Of the 48 FoI variables in the test cohort, NLP extracted 43 (90%) and 38 (79%) FoIs with an F-score of at least 0.90 and 0.95 respectively. In comparison, of the same 48 FoI outcome variables, a trained human annotator extracted 44 (92%) and 41 (85%) variables with an F-score of at least 0.90 and 0.95 respectively.

Our NLP system agrees with and extends the NLP strategies previously described by enhancing transparency, scalability, and adaptability [11–15, 31–36]. Ashish et al. developed enhancements to an academic institutional information extraction system to expand the data fields of interest for automated capture of data from cancer pathology reports [12]. The institutional system was built leveraging the existing Unstructured Information Management Architecture framework, resources from the Open Health Natural Language Processing consortium. A Pathology Extraction Pipeline was built upon the established Medical Knowledge Analysis Tool pipeline, which focuses on pathology reports. Xie et al. utilized the Text Information Extraction System to identify potential new cancer diagnoses in real-time using concept terms from the National Cancer Institute Metathesaurus and codes to identify breast cancer from the Unified Medical Language System Terminology Service [31]. Although promising in their application, these software solutions have rigid algorithms that lack the capacity to customize outcome variables of interest, data source formats, and scripts. With the rapidity of novel computational method development, it is essential to ensure the programming of the algorithm can be updated with advances both in NLP strategies and, more importantly, in clinical research [31–36]. Adapting and extending such tools is computationally challenging, limiting customization to meet institutional needs and extensibility to ensure long-term use.

Overall, our NLP pipeline has three main advantages compared to existing solutions: 1) robust encoding performance metrics; 2) high customizability for adapting additional report types as data sources and FoIs; 3) consistent and rapid processing of documents. The word embedding model has a vocabulary size of 785,000 words, which we adopted to circumvent the need for large-scale, manually labeled training data as required by supervised NLP methods. Compared to a “black box” DL approach, embedding vectors can be interpreted by examining their semantic relationships [37, 38]. Compared to a lexicon-based approach, embeddings are more robust to rare words: while lexicon algorithm necessitates a lookup dictionary which may not cover edge case synonyms, embedding vectors can be generated for any extracted word, which we match to its most semantically similar encoding candidate (Appendix B). Given the high levels of test accuracy, our system may serve as a substitute to manual extraction by researchers and clinicians in an end-to-end, fully autonomous manner. The NLP pipeline was successfully developed with a minimal curated dataset to provide users with a scalable and applicable system at an institutional or regional level. With the goal of extending this user-friendly NLP pipeline, the system may be used to help guide human reviewers by recommending encodings, highlighting the source of information to reduce search time by human extractors. For transparency with expanding targeted outcomes of interest, the NLP system FoIs may be altered and easily validated through visual comparisons to optimize the algorithms’ accuracy. Lastly, the NLP system can be indefinitely fine-tuned to prevent reoccurring errors. With the increasing investment of both data and time, this iterative adaptation of the system allows the pipeline accuracy to asymptotically approach, or even exceed, expert human accuracies.

With the NLP pipeline accuracy above 90% for salient outcomes in breast cancer research, this system may be applied in clinical population-based research studies. For example, this system may be applied to provide timely reports on cancer outcomes in comparative effectiveness studies following the approval of a new treatment modality. It may also be used for rapid assessment of implementation following release of clinical guidelines.

Limitations of the NLP system are inherent in the nature of the data extracted. Error analysis revealed that some difficulties could be attributed to unexpected signpost phrase variations, which may be resolved with larger training datasets to cover these edge cases. Some other errors resulted from the industrial OCR module, which converts the scanned image to text incorrectly. Auto-correction mechanisms may be a solution, relying on the iterative growth of the ontology. Note that the OCR step can be skipped (and thus eliminate many issues) if the NLP system has access to reports in raw text file format. Although the use of a minimal dataset is a strength of the developed pipeline, the smaller subset of curated information may limit the encodings of the algorithm, leading to disagreements between the NLP system and human annotator. Fortunately, the pipeline can rapidly adopt new FoIs with the addition of a custom word embedding function without the need for thousands of reports. For example, text such as “cannot be determined – larger than 10 mm” could be embedded near the “10 mm cluster”, rather than the “N/A cluster”, or vice versa.

Lastly, the current pipeline cannot perform data extraction with sufficient levels of accuracy with completely unstructured data. Further work is underway to analyze unstructured text in the EHR by applying and augmenting the NLP strategies of auto-correction, document segmentation, name-entity recognition, tagging, syntactic parsing, information extraction, and classification. Integrating these approaches with the current workflow will improve the generalizability, robustness, and interpretability of the system.

## Conclusion

The NLP system successfully extracts targeted outcome variables to serve as a fruitful data source for downstream clinical research. This system uniquely provides a robust solution for transparent, adaptable, and scalable data automation using minimized sources of curated medical information.

## Supplementary Information

**Additional file 1.**

**Additional file 2.**

**Additional file 3.**

**Additional file 4.**

## Data Availability

Individual participant data will be available (including data dictionaries). Individual participant data that underlie the results reported in this article, after de-identification will be made available (text, tables, figures, and appendices), including the study protocol. Data sharing will occur beginning 6 months and ending 12 months following article publication. Data sharing will occur with investigators whose proposed use of the data has been approved by an independent review committee identified for this purpose and who provide a methodologically sound proposal as reviewed by the authors. Data sharing will be for analyses required by the approved proposal. Proposals may be submitted up to 12 months following article publication. After 12 months, the data will be available in our institution’s data warehouse but without investigator support other than deposited metadata. Requests for submitting proposals and accessing data should be addressed to the corresponding author, Dr. Kathryn V. Isaac.

## Abbreviations

NLP: Natural Language Processing
EHR: Electronic health record
NER: Named entity recognition
IE: Information extraction
TC: Text classification
DL: Deep learning
OCR: Optical Character Recognition
FoI: Field of Interest
GT: Ground Truth
DCIS: Ductal Carcinoma In Situ disease
