# Supplementary material for: Automated medical chart review for breast cancer outcomes research: a novel natural language processing extraction system
Source: BMC Med Res Methodol. 2022 May 12;22:136. doi: 10.1186/s12874-022-01583-z (PMC9101856; doi:10.1186/s12874-022-01583-z)
Supplement: Supplementary file 1 — Additional file 1. [file 12874_2022_1583_MOESM1_ESM.docx]

Supplemental Table 1. Outcome variables defined according to codebook. IR: Immediate reconstruction; ADM: Acellular Dermal Matrix; IMF: Inframammary fold; LN: lymph node. For all “Other” classifications, values were specified in raw text.

| **Operative report variables** | |
| --- | --- |
| Laterality | 1=Left 2=Right 3=Bilateral |
| Surgical Indication | 1=Primary treatment 2=Re-excision of positive margins after BCS 3=Completion mastectomy after BCS with positive margins 4=Recurrent cancer 5=Second primary |
| Pre-Operative Biopsy | 0=None 1=Core biopsy 2=Surgical biopsy |
| Pre-Operative Diagnosis | 1=Invasive carcinoma 2=DCIS 3=Invasive carcinoma and DCIS 4=Multicentric breast cancer 5=DCIS with microinvasion |
| Neoadjuvant Treatment | 0=None 1=Chemotherapy 2=Chemotherapy and Radiation |
| Breast Procedure | 1=Lumpectomy/partial mastectomy 2=Nipple-sparing mastectomy 3= Skin-sparing mastectomy 4=Total mastectomy |
| Immediate IR | 0=No 1=Yes |
| IR Type | 0=Not mentioned 1=Level 3 Oncoplastic closure 2=Tissue Expander+ADM 3=Implant+ADM 4=DIEP 5=Other Autologous 6=Fat grafting 7=Tissue Expander Only 8=Implant Only 9=Other |
| Wire Localization | 1=Yes 2=No |
| Incision Type | 1= Curvilinear 2=Elliptical 3=Radial 4=IMF 5=Peri/circum-areolar w/ lateral extension 6=Peri/circum-areolar w/ inferior vertical extension+lateral IMF extension 7=Superior aspect of Wise 8=Inferior aspect of Wise 9=Keyhole 10=Other |
| Axillary Surgery | 0=None 1=Sentinel Lymph Node Biopsy 2=Axillary Lymph Node Dissection |
|  | |
| **Pathology report variables** | |
| Breast Lesion Pathologic Diagnosis | |
| Invasive Carcinoma | 0=Absent 1=Present |
| Invasive Histologic Type | 0=N/A 1=Lobular 2=Ductal 3=Mucinous 4=Tubular 5=Micro-invasive carcinoma 6=Other |
| Nottingham Score (total) | 0=N/A 3=3 4=4 5=5 6=6 7=7 8=8 9=9 |
| *Glandular Differentiation* | 0=N/A 1=1 2=2 3=3 |
| *Nuclear Pleomorphism* | 0=N/A 1=1 2=2 3=3 |
| *Mitotic Rate* | 0=N/A 1=1 2=2 3=3 |
| Histologic Grade | 0=N/A 1=I 2=II 3=III |
| Tumour Size | mm |
| Tumour Focality | 0=N/A 1=Single 2=Multifocal |
| # of Foci | 0=not specified #=# |
| Tumour Site | clock orientation |
| Lymphovascular Invasion | 0=Absent 1=Present 2=Cannot be determined |
| In situ Component | 0=Absent 1=Present |
| In situ Type | 0=N/A 1=Lobular 2=Ductal 3=Lobular and ductal 4=Other |
| In situ Nuclear Grade | 0=N/A 1=I 2=II 3=III |
| Necrosis | 0=Not identified 1=Present |
| Architectural Patterns | 1= Comedo, 2= cribriform, 3= micropapillary, 4=Other |
|  | |
| Surgical margins | |
| Invasive Carcinoma Margins | 0=negative 1=positive 2=Can't be assessed |
| Distance from Closest Margin | mm |
| Closest Invasive Margin location | 0=N/A 1=Posterior 2=Anterior 3=Lateral 4=Medial 5=Anterior and posterior 6=Inferior 7=Superior 8=Other |
| DCIS Margins | 0=negative 1=positive |
| Distance of DCIS from Closest Margin | mm |
| Closest DCIS Margin location | 0=N/A 1=Posterior 2=Anterior 3=Lateral 4=Medial 5=Anterior and posterior 6=Inferior 7=Superior 8=Other |
|  | |
| Lymph Nodes | |
| Total LN Examined | # |
| # Sentinel LN | # |
| Micro/macro metastasis | 0=Absent 1=Present |
| # LN w/ Micrometastasis | # |
| # LN w/ Macrometastasis | # |
| Size of Largest Macrometastasis Deposit | mm |
| Extranodal Extension | 0=Absent 1=Present |
| Extent | mm |
|  | |
| Pathologic Stage | |
| Tumour Size | mm |
| # Sentinel Nodes | # |
| # Micrometastatic Nodes | # |
| # Macrometastatic Nodes | # |
| Pathologic Stage | TNM |
