# Supplementary material for: Automated medical chart review for breast cancer outcomes research: a novel natural language processing extraction system
Source: BMC Med Res Methodol. 2022 May 12;22:136. doi: 10.1186/s12874-022-01583-z (PMC9101856; doi:10.1186/s12874-022-01583-z)
Supplement: Supplementary file 2 — Additional file 2. [file 12874_2022_1583_MOESM2_ESM.docx]

| Field of Interest | Accuracy | Same | Different | Missing |
| --- | --- | --- | --- | --- |
| Laterality | 0.90 | 46 | 3 | 2 |
| Surgical Indication | 0.92 | 47 | 2 | 2 |
| Pre-Operative Biopsy | 0.96 | 49 | 0 | 2 |
| Pre-Operative Diagnosis | 0.92 | 47 | 3 | 1 |
| Neoadjuvant Treatment | 0.98 | 50 | 0 | 1 |
| Breast Procedure | 0.76 | 39 | 11 | 1 |
| Immediate Reconstruction Mentioned | 0.90 | 46 | 4 | 1 |
| Immediate Reconstruction Type | 0.75 | 38 | 11 | 2 |
| Wire Localization | 0.86 | 44 | 5 | 2 |
| Breast Incision Type | 0.88 | 35 | 2 | 3 |
| Axillary Surgery | 0.92 | 47 | 1 | 3 |
| **Overall** | **0.887** | **488** | **42** | **20** |

Supplemental Table 2: Preliminary performance of NLP system extraction with respect to the ground truth (GT) in operative reports (A) and pathology report (B).

A) Operative report variables:

B) Pathology report variables:

| Field of Interest | Accuracy | Same | Different | Missing |
| --- | --- | --- | --- | --- |
| Invasive Carcinoma | 0.96 | 49 | 2 | 0 |
| Invasive Histologic Type | 0.82 | 42 | 9 | 0 |
| Nottingham Score | 0.96 | 49 | 1 | 1 |
| Glandular Differentiation | 0.94 | 48 | 2 | 1 |
| Nuclear Pleomorphism | 0.98 | 50 | 1 | 0 |
| Mitotic Rate | 0.98 | 50 | 1 | 0 |
| Histologic Grade | 0.96 | 47 | 1 | 1 |
| Tumour Size (mm) | 0.92 | 47 | 2 | 2 |
| Tumour Focality | 0.98 | 48 | 1 | 0 |
| # of Foci | 0.94 | 47 | 0 | 3 |
| Tumour Site | 1.00 | 47 | 0 | 0 |
| Lymphovascular Invasion | 0.98 | 50 | 1 | 0 |
| In-situ Component | 0.96 | 48 | 2 | 0 |
| In-situ Type | 0.75 | 36 | 11 | 1 |
| In-situ Nuclear Grade | 1.00 | 50 | 0 | 0 |
| Necrosis | 0.98 | 50 | 1 | 0 |
| DCIS Extent | 0.69 | 35 | 0 | 16 |
| Architectural Patterns | 1.00 | 51 | 0 | 0 |
| Invasive Carcinoma Margins | 0.96 | 48 | 2 | 0 |
| Distance from  Closest Margin | 0.84 | 43 | 1 | 7 |
| Closest Margin | 0.90 | 43 | 1 | 4 |
| DCIS Margins | 0.76 | 35 | 11 | 0 |
| Distance of DCIS from  Closest Margin (mm) | 0.86 | 42 | 2 | 5 |
| Closest Margin DCIS location | 0.84 | 41 | 2 | 6 |
| Total LN Examined | 0.98 | 49 | 0 | 1 |
| # Sentinel LN Examined | 1.00 | 50 | 0 | 0 |
| Micro/macro metastasis | 0.90 | 46 | 5 | 0 |
| # LN w/ Micro-metastasis | 1.00 | 51 | 0 | 0 |
| # LN w/ Macro-metastasis | 1.00 | 51 | 0 | 0 |
| Size of Largest  Macro-metastasis Deposit | 0.98 | 50 | 0 | 1 |
| Extranodal Extension | 1.00 | 51 | 0 | 0 |
| Extent (mm) | 1.00 | 47 | 0 | 0 |
| Invasive Tumour Size (mm) | 0.94 | 45 | 2 | 1 |
| # Sentinel Nodes Examined | 0.96 | 45 | 2 | 0 |
| # Micro-metastatic Nodes | 1.00 | 50 | 0 | 0 |
| # Macro-metastatic Nodes | 1.00 | 51 | 0 | 0 |
| Pathologic Stage | 0.98 | 50 | 0 | 1 |
| **Overall** | **0.938** | **1732** | **63** | **51** |
