# Supplementary material for: Automated medical chart review for breast cancer outcomes research: a novel natural language processing extraction system
Source: BMC Med Res Methodol. 2022 May 12;22:136. doi: 10.1186/s12874-022-01583-z (PMC9101856; doi:10.1186/s12874-022-01583-z)

Appendix A

A 3D visualization of codebook embedding vectors. Similar encodings words are clustered together. The robustness of embedding vectors is shown by linear word analogies: For example, on the right-most side of this plot, an imaginary line between “DCIS” and “LCIS” would be parallel to a line between “ductal” and “lobular.”


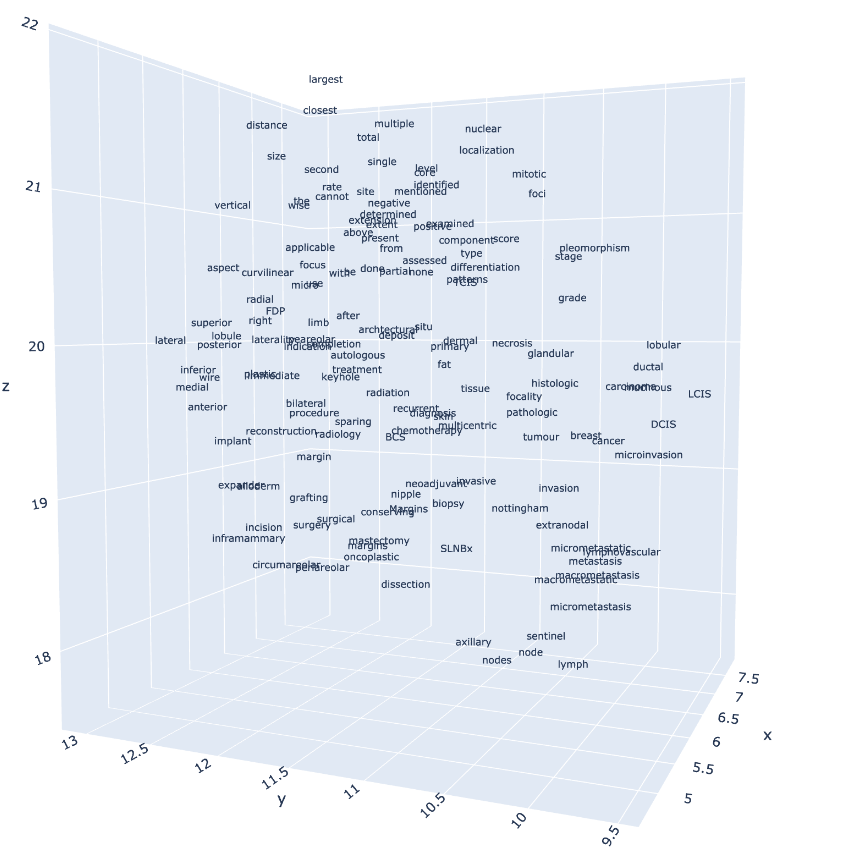

Supplement: Supplementary file 3 — Additional file 3. [file 12874_2022_1583_MOESM3_ESM.docx]
