# Supplementary material for: Automated medical chart review for breast cancer outcomes research: a novel natural language processing extraction system
Source: BMC Med Res Methodol. 2022 May 12;22:136. doi: 10.1186/s12874-022-01583-z (PMC9101856; doi:10.1186/s12874-022-01583-z)

Appendix B

An illustration of the embedding-based encoding algorithm. In this example, the word “lobule” was extracted from the EHR, and its embedding vector is compared to the codebook candidates’ embeddings using cosine similarity. The NLP pipeline chooses to encode “lobular” which gives the highest similarity score.


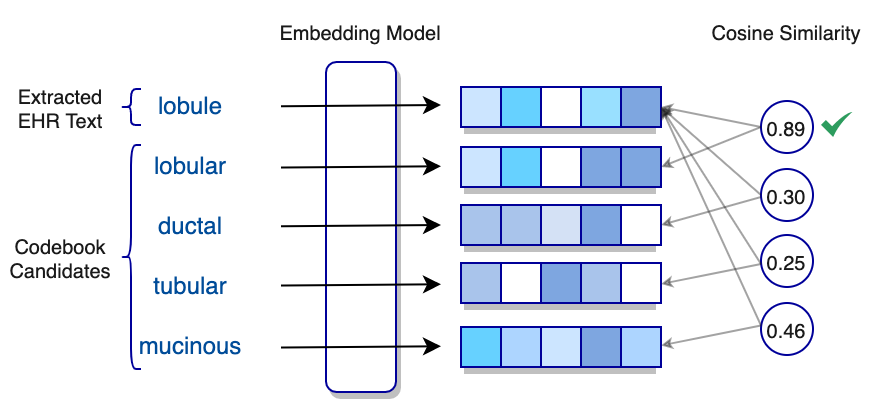

Supplement: Supplementary file 4 — Additional file 4. [file 12874_2022_1583_MOESM4_ESM.docx]
